# miR-27a-containing Exosomes Secreted by Irradiated Skin Keratinocytes Delayed the Migration of Unirradiated Skin Fibroblasts

**DOI:** 10.7150/ijbs.35356

**Published:** 2019-08-19

**Authors:** Wen Tan, Yarui Zhang, Mengting Li, Xueting Zhu, Xuejiao Yang, Jingdong Wang, Shuyu Zhang, Wei Zhu, Jianping Cao, Hongying Yang, Liyuan Zhang

**Affiliations:** 1 State Key Laboratory of Radiation Medicine and Protection, School of Radiation Medicine and Protection, Medical College of Soochow University/Collaborative Innovation Center of Radiation Medicine of Jiangsu Higher Education Institutions, 199 Renai Road, Suzhou Industrial Park, Suzhou, Jiangsu Province, 215123, P. R. China; 2 Department of Radiotherapy and Oncology, The Second Affiliated Hospital of Soochow University, Institute of Radiotherapy & Oncology, Soochow University, 1055 Sanxiang Road, Suzhou, Jiangsu Province, 215004, P. R. China

**Keywords:** radiation-induced bystander effect, intercellular communication, cell migration, miR-27a, exosomes, ROS

## Abstract

Radiation-induced bystander effect (RIBE), e.g. the biological response occurring in unirradiated cells when their neighboring cells are irradiated, is the consequence of intercellular communication between irradiated and unirradiated cells and intracellular signal transduction of these two cell populations. Although several miRNAs have been found to play an important role in RIBEs, the evidence for the regulatory effects of miRNAs on RIBEs is still limited. In this study, by using a two cell-line co-culture system, we first found that the migration of unirradiated bystander WS1 skin fibroblasts was inhibited after co-culture with irradiated HaCaT skin keratinocytes. Further study revealed that HaCaT cells exposed to α-particles and X-rays quickly showed an elevated miR-27a expression, which was essential for the induction of the bystander effect, resulting in the secretion of miR-27a-containing exosomes as a major RIBE signaling factor. Upon uptake of these exosomes, the recipient unirradiated WS1 cells displayed oxidative stress and increased miR-27a levels. Elevated levels of miR-27a that targets MMP2 in the recipient WS1 cells then led to slowed cell migration, which was dependent upon the redox status of WS1 cells. To summarize, the present study has revealed a critical role of miR-27a in every step of the induction of bystander migration inhibition of unirradiated WS1 fibroblasts co-cultured with irradiated HaCaT keratinocytes, confirming the important regulatory effects of miRNAs in RIBEs. Additionally, we provided direct evidence that RIBEs could affect wound healing.

## Introduction

Radiation-induced bystander effect (RIBE) refers to the biological response occurring in unirradiated cells when their neighboring cells are irradiated. It may manifest as DNA damage, micronucleus induction, gene mutation, change in gene expression, genomic instability, cell killing, etc. 1-6. RIBEs have attracted great interest from radiation biologists and radiation oncologists since they may play an important role in both health risk of radiation at low doses and efficacy and side effects of radiotherapy at high doses 7-9. Moreover, RIBEs are indeed the consequence of intercellular communication between irradiated and unirradiated cells and intracellular signal transduction of these two cell populations, thus the underlying mechanisms of RIBEs may also be of interest to researchers from other fields. So far, intercellular gap junction 2, 10, soluble signaling molecules such as cytokines 11-14, cysteine protease CPR-4 15, etc. have been found to mediate the communication between irradiated and unirradiated cells. Radiation-induced signaling pathways such as iNOS-NO signaling 16, TGF-β1 signaling 17, etc. in irradiated cells, stress-induced signaling pathways such as p38 pathway 16, TGF-β1 signaling 17, etc., as well as reactive oxygen species (ROS) 3, 18, 19 in unirradiated bystander cells have also been demonstrated to play important roles in the occurrence of RIBEs.

Epigenetic effectors including miRNAs were proposed to be one of the potential mechanisms underlying RIBEs 20, since an induction of epigenetic changes such as alterations in DNA methylation, histone methylation and miRNA expression had been observed in bystander tissues or organs 21, 22. However, by depleting mature miRNAs, Dickey et al. did not observe any difference in bystander DNA damage, thus they concluded that miRNAs might not be the primary bystander signaling molecules in RIBE, instead changes in miRNAs were more likely to be a bystander effect manifestation 23. In contrast to their conclusion, our previous studies and the studies from other groups clearly demonstrated that certain miRNAs such as miR-21, miR-1246, miR-7-5p, miR-663, etc. played an important regulatory effect on RIBEs 17, 24-30, supporting the hypothesis that miRNAs may act as major bystander effectors. According to these studies, miRNAs can mediate RIBEs not only via regulating the signaling in irradiated cells and/or bystander cells 17, 26-29, but also through serving as bystander signaling molecules mediating intercellular communication between irradiated and bystander cells 24, 25, 30. These bystander signaling molecules have been found to be encapsulated into the exosomes released by irradiated cells, and the uptake of the exosomes by unirradiated cells thus resulted in bystander effects 24, 25, 30.

Exosomes, one kind of extracelluar vesicles with a typical size of 40-100 nm, which were once regarded as a means of disposing cellular waste, have recently become a hot topic since they have been found to be able to transport their cargo such as nucleic acids, proteins and lipids between cells thus mediating intercellular communication 31. There is evidence for that ionizing radiation (IR) induces exosome secretion in both dose and time dependent manners 32. Moreover, IR causes changes in exosome composition such as up- and down-regulation of some specific mRNAs and proteins 32-36. Limited literature data have also shown radiation-induced changes in some specific miRNAs in exosomes 25, 37. Importantly, RIBEs can be mediated by miRNA-containing exosomes 24, 25, 30. In spite of all those studies, the evidence for the involvement of miRNAs in RIBEs and the underlying mechanisms are still limited.

In this study, by using a two-cell line co-culture system e.g. irradiated HaCaT skin keratinocytes and unirradiated WS1 skin fibroblasts, we investigated the roles of miRNAs in RIBEs. We found that unirradiated WS1 cells were under oxidative stress and migrated more slowly after co-culture with irradiated HaCaT cells. We identified miR-27a as an important mediator for bystander effects. HaCaT cells showed up-regulation of miR-27a after exposed to both alpha particles and X-rays, then released miR-27a-containing exosomes into co-culture system. When unirradiated bystander WS1 cells took up the exosomes, their intracellular ROS levels increased, and their miR-27 expression was also up-regulated, then resulted in down-regulation of its target, MMP2, thus leading to slowed cell migration. Furthermore, the exosomes extracted from irradiated HaCaT cells inhibited wound healing *in vivo*. These data suggest that miR-27a mediates RIBEs in bystander WS1 cells by regulating every step involved in the induction of RIBEs. Moreover, the bystander migration inhibition seems to be dependent upon the redox status of unirradiated WS1 cells. These data may have an application in radiation protection in environmental, occupational and clinical scenarios.

## Materials and Methods

### Cells and co-culture system

A transwell insert co-culture system was utilized to study medium-mediated bystander effects in unirradiated WS1 fibroblasts induced by irradiated HaCaT keratinocytes, as previously described 27. Briefly, WS1 cells were seeded into the wells of 6-well plates and HaCaT cells were seeded on the coverslips in companion Millicell^®^ transwell culture inserts that fit 6-well plates (Millipore, MA, USA). Immediately after radiation, inserts with irradiated HaCaT cells were put into wells with unirradiated WS1 cells, so that irradiated HaCaT cells and unirradiated WS1 cells were 3 mm apart, but shared the same medium through the porous membrane of inserts with a pore size of 0.4 μm to allow the passage of molecules but not cells.

WS1 cells stably overexpressing SOD2 and the relative control cells were also used. After transfected with pCMV-SOD2 and pCMV plasmids (Sino Biological Inc. Beijing, China) using Lipofectamine^TM^ 2000 (Invitrogen, Carlsbad, CA, USA), the successfully transfected WS1 cells were selected by hygromycin B for 2-3 weeks (0.2 mg/ml). Then the stably transfected cells were kept in complete medium containing 0.1 mg/ml of hygromycin B.

### Chemical

N-acetyl-L-cysteine (NAC) purchased from Sigma-Aldrich (St. Louis, MO, USA) was used as the ROS scavenger. The working concentration for NAC was 1 mM.

### Cell irradiation

Twenty-four hours after plating, HaCaT cells on coverslips were irradiated at room temperature (RT) with 0.56 Gy of alpha particles using our ^241^Am α-irradiator at a dose rate of 0.14 Gy/min as described previously 38. For X-irradiation, HaCaT cells were replenished with fresh medium, then were irradiated with 1 Gy of 160 kVp X-rays (RAD SOURCERS2000 X-ray machine, USA) at a dose rate of 1.20 Gy/min. 0.56 Gy for alpha particles and 1 Gy for X-rays were used since they caused comparable reduction of cell survival in HaCaT cells 28. The LET values for α particles and X-rays were about 95 and 2 keV/μm, respectively.

### Cell proliferation assay

WST-1 Cell Proliferation and Cytotoxicity Assay Kit (Beyotime, China) was used to evaluate the effect of irradiated HaCaT cells on the proliferation of bystander WS1 fibroblasts according to the manufacture's protocols.

### Wound healing assay

1×10^6^ WS1 fibroblasts were seeded into each well of 6-well plates. After 24 h, the confluent cells were replenished with fresh serum-free culture medium and cultured for 8-10 hours. Then a straight scratch was made in each well using a sterile 200 μl pipette tip, and cells were kept in co-culture with irradiated HaCaT cells or exosomes from irradiated HaCaT cells in fresh medium containing 0.5% FBS. At different times, images of wound scratch were taken under a microscope (DM2700, Leica, Germany). And scratch areas were analyzed by using Image J (NIH, USA).

### Measurement of intracellular ROS

Cellular ROS Detection Assay Kit (Abcam, UK) was used to detect the changes in intracellular ROS levels of WS1 cells. In brief, WS1 cells in suspension were incubated in 2, 7-dichlorfluorescein-diacetate (DCFDA, 20 μM) working medium in the dark at 37 ℃ for 30 min. Then they were put into co-culture with irradiated HaCaT cells or exosomes from irradiated HaCaT cells in phenol red-free DMEM for 1 h at 37 ℃. And 1×10^4^ WS1 cells from each sample were transferred to a 96-well plate suitable for fluorescence measurement. The fluorescence at 528 nm with an excitation wavelength of 485 nm was measured on a fluorometric plate reader (Synergy2, USA). Each sample was measured in triplicate. Cells treated with 50 μM Tert-Butyl Hydrogen Peroxide were used as positive control.

### Isolation and identification of exosomes derived from HaCaT cells

To avoid the influence of fetal bovine serum on exosomes, HaCaT cells were cultured in serum-free medium for 12 hours prior to radiation exposure. Conditioned media were collected at different times post radiation, and cell debris were removed by centrifuging at 2,000 g for 30 min, and then incubated with the Total Exosome Isolation Reagent (4478359, Thermo Fisher, USA) overnight at 4 ℃. After centrifuging at 10,000 g for 1 h, exosome pellets were resuspended in PBS and aliquoted. For electron microscopy, exosomes were stored at 4 ℃, and the other aliquots were stored at -80 ℃. The concentration of exosomes was determined by BCA protein assay kit, as suggested by the manufacturer (Beyotime Institute of Biotechnology, China). The morphology of exosomes derived from unirradiated and irradiated HaCaT cells was observed by transmission electron microscopy (Tecnai G2 Spirit BioTwin, USA). The size distribution of exosomes were measured by Zetasizer Nano ZS90 (Malvern, UK).

### Observation of uptake of exosomes by recipient WS1 cells using fluorescent microscopy

Exosomes were labelled with DiI (Beyotime, China) before adding into WS1 cell culture for 30 min and 2 h. Then the medium containing DiI was discarded, the cells were washed with PBS twice, and fixed with paraformaldehyde for 15 min at RT. After counter stained with DAPI (Beyotime, China), cells were observed and images were taken under a fluorescent microscope (DM2700, Leica, Germany).

### microRNA extraction and Real time PCR

At various time points, irradiated HaCaT cells and bystander WS1 cells were collected, and the microRNA was isolated and purified using the E.Z.N.A.^TM^ miRNA Kit (Omega Bio-Tek Inc., USA). Reverse transcription and quantitative real-time PCR were subsequently performed using the TaqMan^®^ MicroRNA Reverse Transcription Kit and the TaqMan^®^ MicroRNA Assays (AB Applied Biosystems, USA) according to the manufacture's protocols. The PCR results were normalized with the internal control, RNU6B. And the expression of miR-27a/b in the treated cells was expressed as fold changes compared with that in the untreated control.

To determine the quantities of miR27a/b in the exosomes secreted by irradiated and unirradiated HaCaT cells, absolute quantitative real-time PCR was carried out. After reverse transcription of miR27a/b mimics (150 ng/15 µl), the cDNA solution was gradient diluted, which matched to 10, 1, 0.1, 0.01 and 0.001 ng/μl of starting miRNA mimics, and quantitative real-time PCR was subsequently performed to create a standard curve. Thus the quantities of miR27a/b in the exosomes could be determined using the standard curves.

### Luciferase reporter gene assay

The 5'untranslated region (UTR) of MMP2 and its mutated 5'UTR were cloned into GP-Check2 plasmids (GenePharma, Shanghai), named GP-Check2-MMP2 and GP-Check2-mut-MMP2, respectively. Mock plasmids GP-Check2-Mock was used as negative control. 1.5×10^4^ WS1 cells were transfected with GP-Check2-MMP2, GP-Check2-mut-MMP2 and GP-Check2-Mock constructs, respectively, using lipofectamine® 2000 (Life Technologies, USA). Twenty-four hours later, miR-27a-3p mimics or its negative control (NC) was transfected into the WS1 cells. Luciferase activity was measured on a EnSpire plate reader (Synergy 2, Bio-Tek, USA) 24 h post transfection using a Dual-Glo^®^ luciferase assay system kit (Promega) according to the manufacturer's protocol.

### ShRNA knockdown

Lentiviral particles with three MMP2-targeting shRNA constructs, e.g. sh1 (top strand: GATCCGCAGGTGATCTTGACCAGAATACCATTTCAAGAGAATGGTATTCTGGTCAAGATCACCTGTTTTTTC, Bottom strand: AATTGAAAAAACAGGTG ATCTTGACCAGAATACCATTCTCTTGAAATGGTATTCTGGTCAAGATCACCTGCG), sh2 (top strand: GATCCGCCTTCTTGTTCAATGGCAAGGAGTATTCAAGAGATACTCCTTGCCATTGAACAAGAAGGTTTTTTC, Bottom strand: AATTGAAAAAACCTTCTTGTTCAATGGCAAGGAGTATCTCTTGAATACTCCTTGCCATTGAACAAGAAGGCG), sh3 (top strand: GATCCGCAGGGAATGAA TACTGGATCTACTTTCAAGAGAAGTAGATCCAGTATTCATTCCCTGCTTTTTTC; Bottom strand: AATTGAAAAAAGCAGGGAATGAATACTGGATCTACTTCTCTTGAAAGTAGATCCAGTATTCATTCCCTGCG) and their scramble control shRNA, e.g. NC (top strand: GATCCGTTCTCCGAACGTGTCACGTAATTCAAGAGATTACGTGACACGTTCGGAGAATTTTTTC, Bottom strand: AATTGAAAAAATTCTCCGAACGTGTCACGTAATCTCTTGAATTACGTGACACGTTGGAGAACG) were purchased from Hanbio Biotechnology (Shanghai, China). At 70% confluency, WS1 cells were cultured with the mixture of lentiviral shRNA and polybrene (6 μg/ml, Hanbio Biotechnology, China) for 24 h, then replenished with fresh medium and incubated for another 80 h. Then the cells were validated with qRT-PCR for MMP2 mRNA level (forward primer, ACCCATTTACACCTACACCAAG, reverse primer, CCAAGGTCAATGTCAGGAGAG) for the efficiency of knockdown, and used for the wound scratch assay.

### Western blot analysis and antibodies

The expression of SOD2 and MMP2 protein was detected by western blotting. Briefly, WS1 cells were lysed in lysis buffer (R0020, Solarbio, China) containing 1 mM phenylmethylsulfonyl floride (PMSF, Beyotime, China) to get total proteins. After separated on a 12% or 10% SDS-polyacrylamide gel, the proteins were transferred to a polyvinylidene difluoride membrane (PVDF, BioRad, Hercules, CA, USA). The blots were then probed with rabbit anti-SOD2 mAb (1:300, Boster, Wuhan, China) or rabbit anti-MMP2 mAb (1:1000, Abcam, UK), and mouse anti-β-actin mAb (1:1000, Beotime, China) followed by goat anti-rabbit IgG-horse rabbit peroxidase-conjugated (HRP) antibodies (1: 1000, Beyotime, China) and goat anti-mouse IgG HRP anitbodies (1: 1000, Beyotime, China), respectively. The proteins of interest were chemiluminescently visualized on Typhoon 9410 high performance gel and blot imager (GE Amersham, USA) after treatment of the membrane with ECL kit (Beyotime, China). β-actin was used as loading control. The quantification of the relative expression of MMP2 (expressed as the ratio of MMP2 to β-actin) was performed by using Image J (NIH, USA).

### Mouse skin wound treatment with exosomes

All animal procedures were performed in accordance with Soochow University Medical Experimental Animal Care Guidelines based on the National Animal Ethical Policies. Adult male Balb/c mice (6-8 weeks) were purchased from the Animal Centre of Soochow University. A rectangle wound (1 cm×1.5 cm) was created on each side of the back of mice. Immediately after wound creation, the wound on the left back of a mouse was subcutaneously injected with 200 μg exosomes secreted by X-irradiated HaCaT cells in 200 μl PBS and that on its right back was injected with the same amount of exosomes from sham-irradiated cells. The wound healing process was then observed daily, wound areas were measured by vernier caliper on day 0, 3, 7 and 10 after treatment and calculated. All the mice were sacrificed on day 10 post-surgery, and the tissues of wound area were sectioned for H&E staining.

### *In vivo* tracking

Exosomes were labelled with DiI (20 μM, Beyotime, China) for 15 min at 37 ℃ according to the manufacturer's protocol. Labelled and unlabelled exosomes in PBS were subcutaneously injected into each side of a BALB/c mouse's back with a 1 cm×1.5 cm dorsal wound. Mice were anesthetized and observed under bioluminescence system (IVIS SpectrumCT Small Animal Live Imager, PerkinElmer, USA) on day 0, 3, 7 and 10 after injection, and fluorescence images for exosome distribution were acquired with 549 nm excitation and 565 nm emission filters and analyzed with Living image (Spectrum, Germany).

### Statistical analysis

All data in this paper are presented as the average of at least three independent experiments ± standard error (SEM). Differences between the control group and the treated group were analyzed using the Student's t test of Origin 8 software. A P value of <0.05 between groups was considered significantly different.

## Results

### Irradiated HaCaT keratinocytes inhibit the migration of unirradiated bystander WS1 fibroblasts, which involves ROS

**Figure 1:**
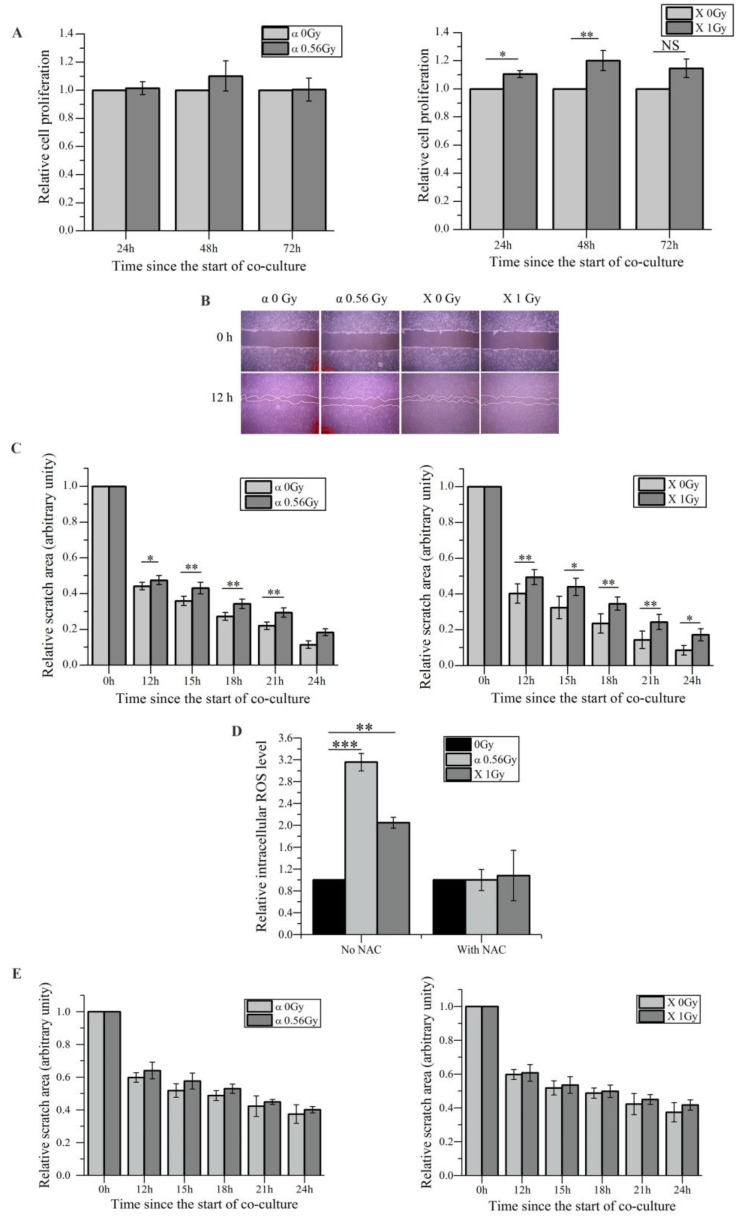
Irradiated HaCaT cells cause slower migration of unirradiated WS1 fibroblasts after co-culture, which involves reactive oxygen species (ROS). (A) The cell proliferation of unirradiated bystander WS1 cells was not inhibited after co-culture with α-irradiated (left panel) and X-irradiated (right panel) HaCaT cells. (B) The representative images of the wound scratches of WS1 cells after co-culture with irradiated HaCaT cells. (C) The quantification of the area of the wound scratches of bystander WS1 cells after co-culture with α-irradiated (left panel) and X-irradiated (right panel) HaCaT cells, showing slowed migration of bystander WS1 cells after co-culture with irradiated HaCaT cells. (D) The elevation of the intracellular ROS levels of bystander WS1 cells after co-culture with irradiated HaCaT cells for 1 h, as well as the effect of NAC on the elevation. (E) The quantification of the area of the wound scratches of bystander WS1 cells after co-culture with α-irradiated (left panel) and X-irradiated (right panel) HaCaT cells in the presence of NAC, showing that NAC almost abolished the slowed migration of bystander WS1 cells after co-culture with irradiated HaCaT cells. All the data represent the means ± SEM from three independent experiments (n=3). *P<0.05, **P<0.01 and ***P<0.001 compared with the relative control.

We have previously demonstrated that irradiated HaCaT cells induce RIBEs such as DNA damage, micronucleus formation, etc. in unirradiated WS1 cells through media-mediated signals 27, 28. Since it has been hypothesized that RIBEs may affect wound healing process 39 , and the proliferation and the migration of skin fibroblasts play important roles in wound healing 40, thus in this study we investigated whether irradiated HaCaT cells would affect the proliferation and the migration of bystander WS1 fibroblasts. First we found that after co-culture with HaCaT keratinocytes irradiated with α-particles, unirradiated bystander WS1 fibroblasts did not show any obvious changes in proliferation, while co-culturing with X-irradiated HaCaT cells even slightly accelerated the proliferation in bystander WS1 cells (Figure 1A). However, compared with the corresponding controls, the wound closure of unirradiated WS1 cells was significantly delayed after co-culture with HaCaT cells irradiated with both α-particles and X-rays (Figure 1B, C). Since the proliferation of bystander WS1 cells was not inhibited after co-culture with irradiated HaCaT cells, these wound scratch assay data suggested that irradiated keratinocytes did slow fibroblast migration via bystander signaling *in vitro*.

**Figure 2:**
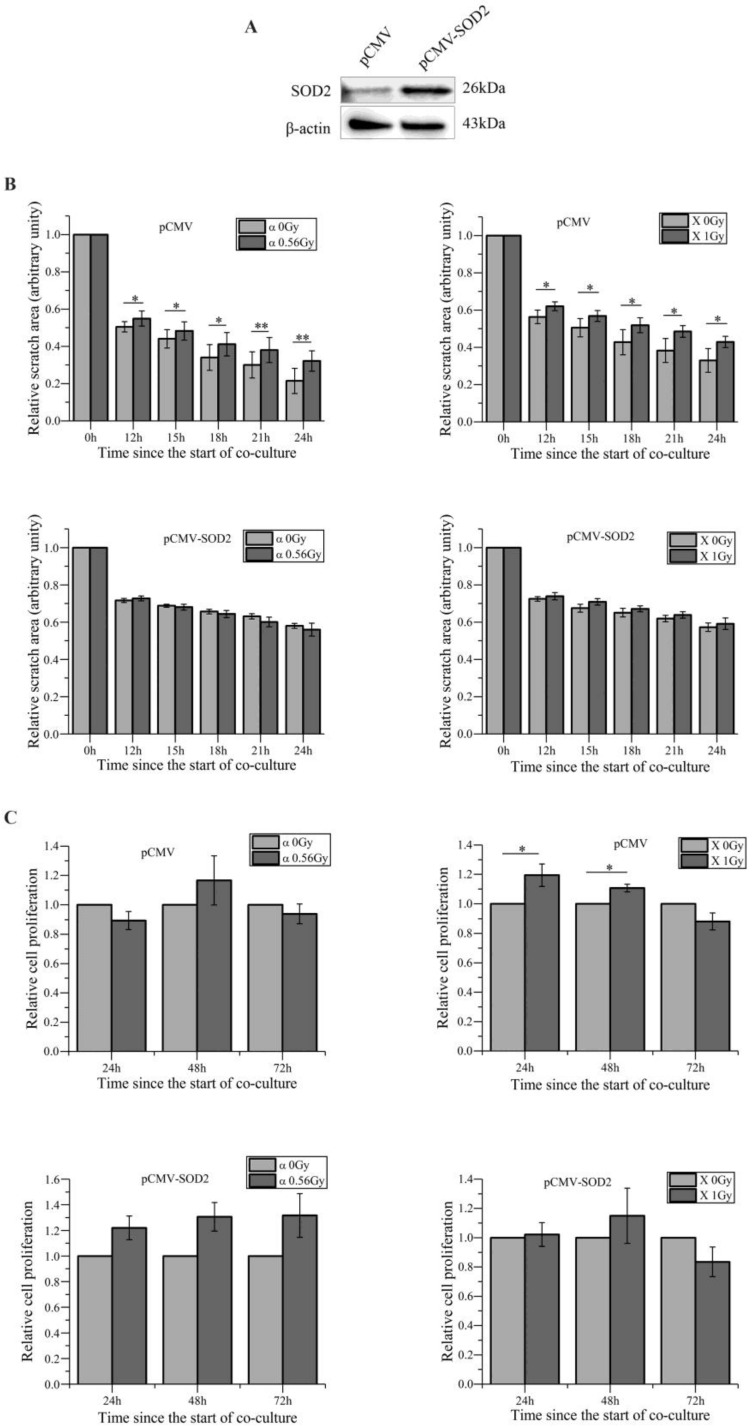
The redox status of bystander WS1 cells affects the bystander inhibited cell migration after co-culture with irradiated HaCaT cells. (A) Western blot image confirming the overexpression of SOD2 in stably-transfected WS1 cells. (B) The quantification of the area of the wound scratches of bystander WS1 cells transfected with pCMV empty plasmids (top panel) and pCMV-SOD2 plasmids (bottom panel) after co-culture with irradiated HaCaT cells. (C) The cell proliferation of bystander WS1 cells transfected with pCMV empty plasmids (top panel) and pCMV-SOD2 plasmids (bottom panel) after co-culture with irradiated HaCaT cells. All the data represent the means ± SEM from three independent experiments (n=3). *P<0.05, and **P<0.01 compared with the relative control.

In addition, we confirmed the increase in the intracellular ROS levels in bystander WS1 cells after co-culture with HaCaT cells irradiated with both α-particles and X-rays (Figure 1D), which we have previously observed 27. When NAC, a ROS scavenger, was added into the co-culture system, the increase in ROS levels was abolished (Figure 1D), and the slowed migration of bystander WS1 cells was also eliminated (Figure 1E). Furthermore, we have previously reported that WS1 cells overexpressing SOD2 no longer showed elevated intracellular ROS levels after co-culture with irradiated HaCaT cells 27. In this study, we found that the migration of WS1 cells overexpressing SOD2 (Figure 2A) was not inhibited after co-culture with irradiated HaCaT cells (Figure 2B). And X-irradiated HaCaT cells also failed to accelerate the proliferation of bystander WS1 cells overexpressing SOD2 (Figure 2C). All these data suggested that ROS played an important role in the bystander effects in unirradiated WS1 cells induced by irradiated HaCaT cells such as enhanced proliferation and slowed migration.

### miR-27a may be responsible for the slowed migration of bystander WS1 cells

**Figure 3:**
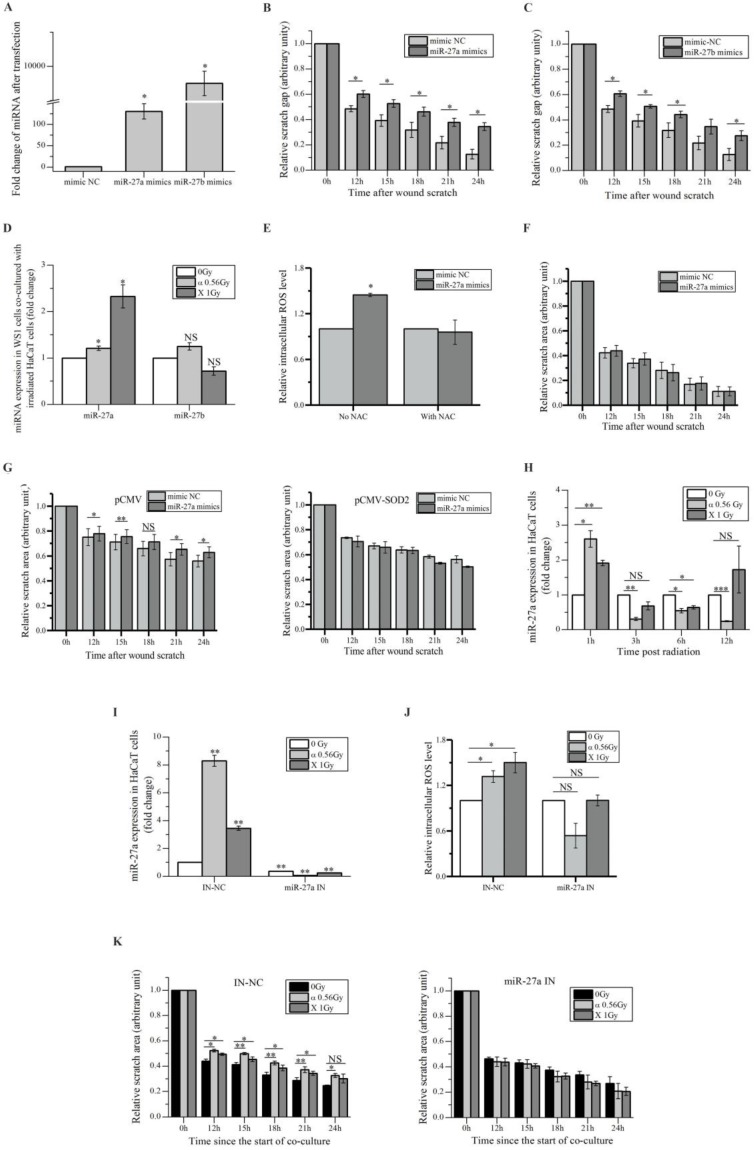
miR-27a is responsible for the slowed migration of bystander WS1 cells. (A) The relative expression levels of miR-27a/b in WS1 cells after transfection with miR-27a/b mimics. (B) The quantification of area of the wound scratches of WS1 cells transfected with miR-27a mimics and negative control (NC), showing that transfection with miR-27a mimics significantly delayed the migration of WS1 cells. (C) The quantification of area of the wound scratches of WS1 cells transfected with miR-27b mimics and NC, showing that transfection with miR-27b mimics significantly delayed the migration of WS1 cells. (D) The alteration of miR-27a/b of bystander WS1 cells after co-culture with irradiated HaCaT cells for 12 h. (E) The relative intracellular ROS levels in WS1 cells transfected with miR-27a mimics and NC in the absence and presence of NAC, showing that NAC abolished the elevation of ROS levels in WS1 cells transfected with miR-27a mimics. (F) The quantification of area of the wound scratches of WS1 cells transfected with miR-27a mimics and NC in the presence of NAC, showing that NAC eliminated the slowed cell migration induced by miR-27a overexpression. (G) The quantification of area of the wound scratches of WS1-pCMV (left panel) and WS1-pCMV-SOD2 (right panel) cells transfected with miR-27a mimics and NC, showing that the redox status of WS1 cells affected the reduction in cell migration rate induced by miR-27a overexpression. (H) The alterations of miR-27a expression levels in irradiated HaCaT cells along with time. (I) The alterations of miR-27a expression levels in irradiated HaCaT cells pre-transfected with miR-27a inhibitors and NC, showing lack of increase in miR-27a expression in HaCaT cells transfected with miR-27a inhibitors after irradiation. (J) The intracellular ROS levels of bystander WS1 cells after co-culture with irradiated HaCaT cells pre-transfected with miR-27a inhibitors and NC, showing that down-regulation of miR-27a in irradiated HaCaT cells abolished oxidative stress in bystander WS1 cells. (K) The quantification of area of the wound scratches of bystander WS1 cells after co-culture with irradiated HaCaT cells pre-transfected with miR-27a inhibitors (right panel) and NC (left panel), showing that down-regulation of miR-27a in irradiated HaCaT cells abolished slowed migration in bystander WS1 cells. All the data represent the means ± SEM from three independent experiments (n=3). *P<0.05, **P<0.01 and ***P<0.001 compared with the relative control. NS represents "not significant".

We aimed to search miRNAs that acted as the bystander mediators. Using miRNA array for preliminary scanning, four miRNAs, e.g. miR-19, miR-27a, miR-27b and miR-141, which have been reported to be involved in cell migration 41-44, were found up-regulated in HaCaT cells irradiated with both α-particles and X-rays (Figure S1A). Since miRNAs can be secreted by cells to induce cell-cell communication 45, to determine what miRNA might be responsible for the slowed migration of bystander WS1 cells co-cultured with irradiated HaCaT cells, we transfected WS1 cells with the relative mimics of these four miRNAs, and found that the cells transfected with the mimics of miR-27a and miR-27b but not miR-19 and miR-141 showed slowed migration (Figure 3A, B, C) and Figure S1B.

Then we determined the changes in the levels of miR-27a and miR-27b in bystander WS1 cells co-cultured with irradiated HaCaT cells. And we observed a significant increase in the levels of miR-27a but not miR-27b in bystander WS1 cells after 12 h of co-culture with HaCaT cells irradiated with both α-particles and X-rays (Figure 3D), the time point at which WS1 cells started to show slowed migration (Figure 1C), suggesting that miR-27a but not miR-27b was responsible for the inhibited WS1 migration. Moreover, the intracellular ROS levels in the WS1 cells transfected with miR-27a mimics were significantly elevated compared with the cells transfected with negative control (NC) (Figure 3E). NAC treatment eliminated the elevation of intracellular ROS levels and the slowed migration of the WS1 cells transfected with miR-27a mimics (Figure 3E, F). Additionally, when the WS1 cells overexpressing SOD2 were transfected with miR-27a mimics, no slowed migration was observed, which was in contrast to the WS1-pCMV cells (Figure 3G). This agreed with the co-culture results described above indicating the involvement of ROS in bystander migration inhibition. All these data suggested an important role of up-regulation of miR-27a in WS1 cells after co-culture with irradiated HaCaT cells in bystander effects.

On the other hand, we confirmed the significant increase in miR-27a levels in HaCaT cells 1 h post irradiation by RT-PCR, followed by a reduction along with time (Figure 3H). To explore the role of miR-27a of irradiated signaling HaCaT cells in RIBEs, we pre-transfected HaCaT cells with miR-27a inhibitors to abolish the increased in miR-27a expression after irradiation (Figure 3I). Then we found that the irradiated HaCaT cells pre-transfected with miR-27a inhibitors failed to cause an increase in intracellular ROS levels (Figure 3J) and a reduction of cell migration rate in bystander WS1 cells (Figure 3K). All these data indicated that miR-27a up-regulation (Figure 3H) in irradiated HaCaT cells shortly after irradiation was essential to the elevation of intracellular ROS levels and the induction of slowed migration of bystander WS1 cells. Thus we hypothesized that miR-27a might be secreted by irradiated HaCaT cells as a RIBE signal.

### miR-27a-containing exosomes secreted by irradiated HaCaT cells mediate bystander effects in unirradiated WS1 cells

**Figure 4:**
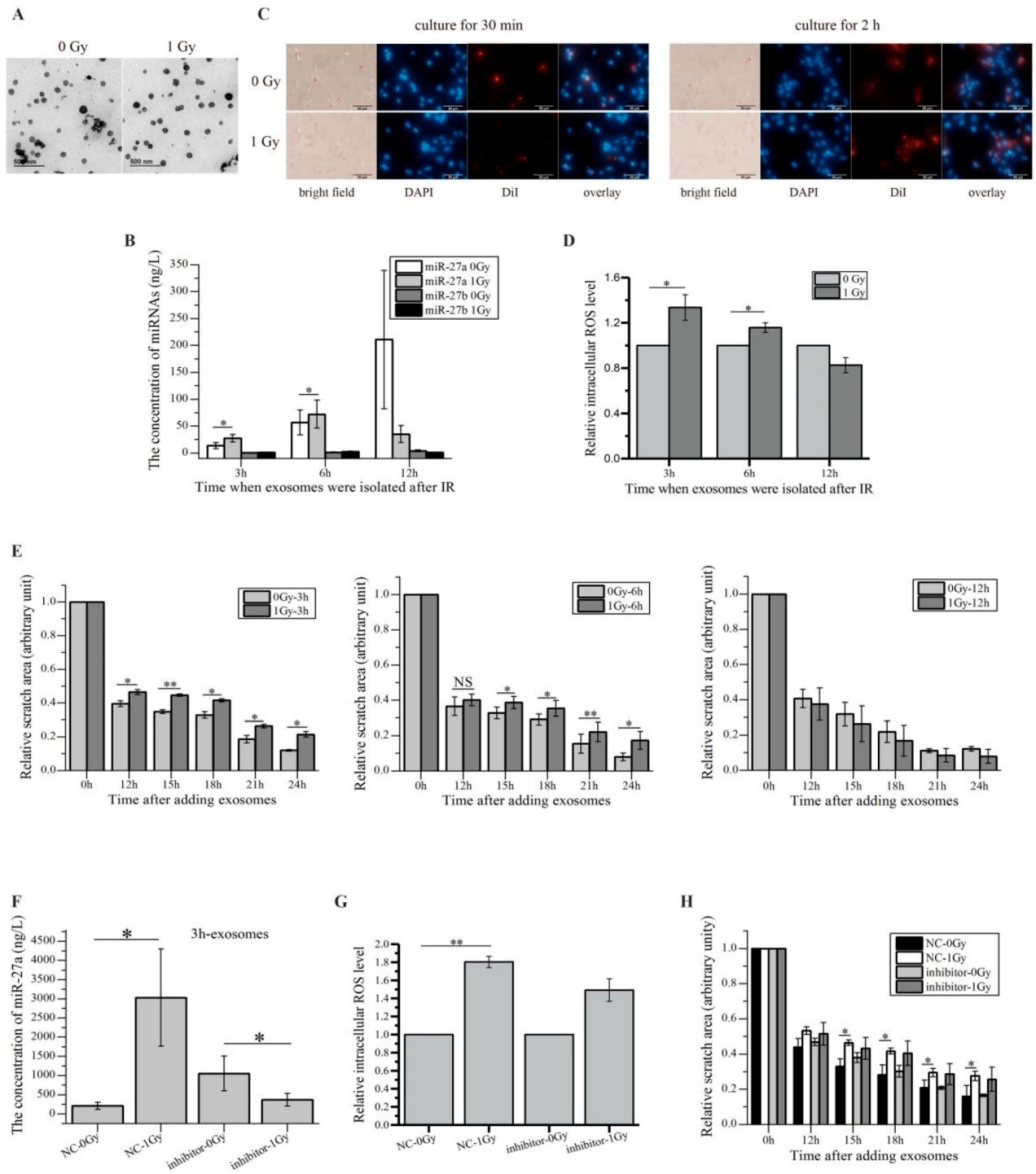
miR-27a-containing exosomes secreted by irradiated HaCaT cells mediate bystander effects in unirradiated WS1 cells. (A) The representative TEM images of the exosomes isolated from media culturing unirradiated and irradiated HaCaT cells. (B) The concentrations of miR-27a/b in the exosomes collected from unirradiated and irradiated HaCaT cells at different times post radiation. (C) Uptake of exosomes from unirradiated and irradiated HaCaT cells by the recipient WS1 cells. Exosomes were pre-labelled with DiI, cell nuclei were stained with DAPI. (D) The alterations of the intracellular ROS levels of the recipient WS1 cells after culture with the exosomes from unirradiated and irradiated HaCaT cells collected at different times post radiation for 1 h, showing that the exosomes from irradiated HaCaT cells collected at 3 and 6 h but not 12 h post radiation caused oxidative stress in the recipient WS1 cells. (E) The quantification of area of the wound scratches of the recipient WS1 cells after culture with the exosomes from unirradiated and irradiated HaCaT cells collected at different times post radiation, suggesting that the exosomes from irradiated HaCaT cells collected at 3 and 6 h but not 12 h post radiation slowed the migration of the recipient WS1 cells. (F) The concentrations of miR-27a in the 3 h exosomes from unirradiated and irradiated HaCaT cells pre-transfected with miR-27a inhibitors and NC, showing lack of increase in miR-27a in the exosomes from irradiated HaCaT cells with down-regulated miR-27a. (G) The alterations of the intracellular ROS levels of the recipient WS1 cells after culture with the 3 h exosomes from unirradiated and irradiated HaCaT cells pre-transfected with miR-27a inhibitors and NC, showing that down-regulated miR-27a in exosomes significantly inhibited the oxidative stress in the recipient WS1 cells. (H) The quantification of the area of the wound scratches of the recipient WS1 cells after culture with the 3 h exosomes from unirradiated and irradiated HaCaT cells pre-transfected with miR-27a inhibitors and NC, showing that down-regulated miR-27a in exosomes significantly inhibited the slowed migration of the recipient WS1 cells. All the data represent the means ± SEM from three independent experiments (n=3). *P<0.05, and **P<0.01 compared with the relative control. NS, not significant.

Since RIBE signals such as miRNAs can be delivered to the recipient cells through exosomes 24, 25, 30, we investigated whether irradiated HaCaT cells secreted miR-27a-containing exosomes to induce RIBEs in unirradiated WS1 cells. Due to the small size of ^241^Am α-particle source (Ø2.0 cm), it was impractical to obtain exosomes from α-irradiated HaCaT cells. Therefore, we only isolated the exosomes from the supernatants of X-irradiated and non-irradiated HaCaT cells. The characteristic cup-shaped morphology of exosomes was confirmed by electron microscopy (Figure 4A). And the size range of the exosomes derived from HaCaT cells showed some changes after radiation exposure (Supple Figure 2), suggesting the difference between the irradiated cells-derived exosomes and non-irradiated cells-derived exosomes.

Moreover, we measured the levels of miR-27a and miR-27b in the exosomes from unirradiated and irradiated HaCaT cells collected at different times post irradiation. The results showed that miR-27b existed in much smaller amount in the exosomes than miR-27a did, and while the miR-27b levels in the irradiated-cell-derived exosomes did not increase significantly compared with those in the unirradiated-cell-derived exosomes, the miR-27a levels in the exosomes from irradiated HaCaT cells collected 3 and 6 h post irradiation increased obviously compared with those in the relevant exosomes from unirradiated cells (Figure 4B). However, no increase in the miR-27a levels was observed when the exosomes were extracted 12 h post radiation (Figure 4B). All these data indicated that irradiated HaCaT cells secreted exosome-encapsulated miR-27a, which was time-dependent. It also agreed with the results above showing that miR-27b might not be an important RIBE factor.

We next examined whether the miR-27a-containing exosomes derived from HaCaT cells could get into the recipient WS1 cells and caused responses. The exosomes from HaCaT cells were labelled with fluorescent DiI, then were added into the culture of WS1 cells, the internalization of exosomes was thus observed (Figure 4C). More importantly, when WS1 cells were cultured with the exosomes collected from irradiated HaCaT cells 3 and 6 h post irradiation for 1 h, the intracellular ROS levels of WS1 cells were significantly elevated compared with the WS1 cells cultured with the exosomes from unirradiated HaCaT cells. But the increase was not observed when WS1 cells were cultured with the exosomes extracted from irradiated HaCaT cells 12 h post irradiation (Figure 4D). Moreover, culturing WS1 cells with the exosomes from irradiated HaCaT cells 3 and 6 h but not 12 h post irradiation also induced significant reduction in WS1 migration rate (Figure 4E).

Additionally, since irradiated HaCaT cells pre-transfected with miR-27a inhibitors could not induced RIBEs in bystander WS1 cells (Figure 3J, K), we examined whether the HaCaT cells with down-regulated miR-27a released miR-27a-containing exosomes upon radiation exposure. And we found no increase in miR-27a levels in the exosomes isolated from irradiated HaCaT cells pre-transfected with miR-27a inhibitors (Figure 4F). Most importantly, the elevation of the intracelluar ROS levels and the slowed cell migration (Figure 4H) of the recipient WS1 cells (Figure 4G) were significantly inhibited after uptake of these exosomes. All these data indicated that the exosomes secreted by irradiated HaCaT cells mediated the increased intracellular ROS levels and delayed migration in the unexposed recipient WS1 cells, and exosome-encapsulated miR-27a was a major signaling molecule.

### miR-27a targets MMP2 to exert bystander effects

**Figure 5:**
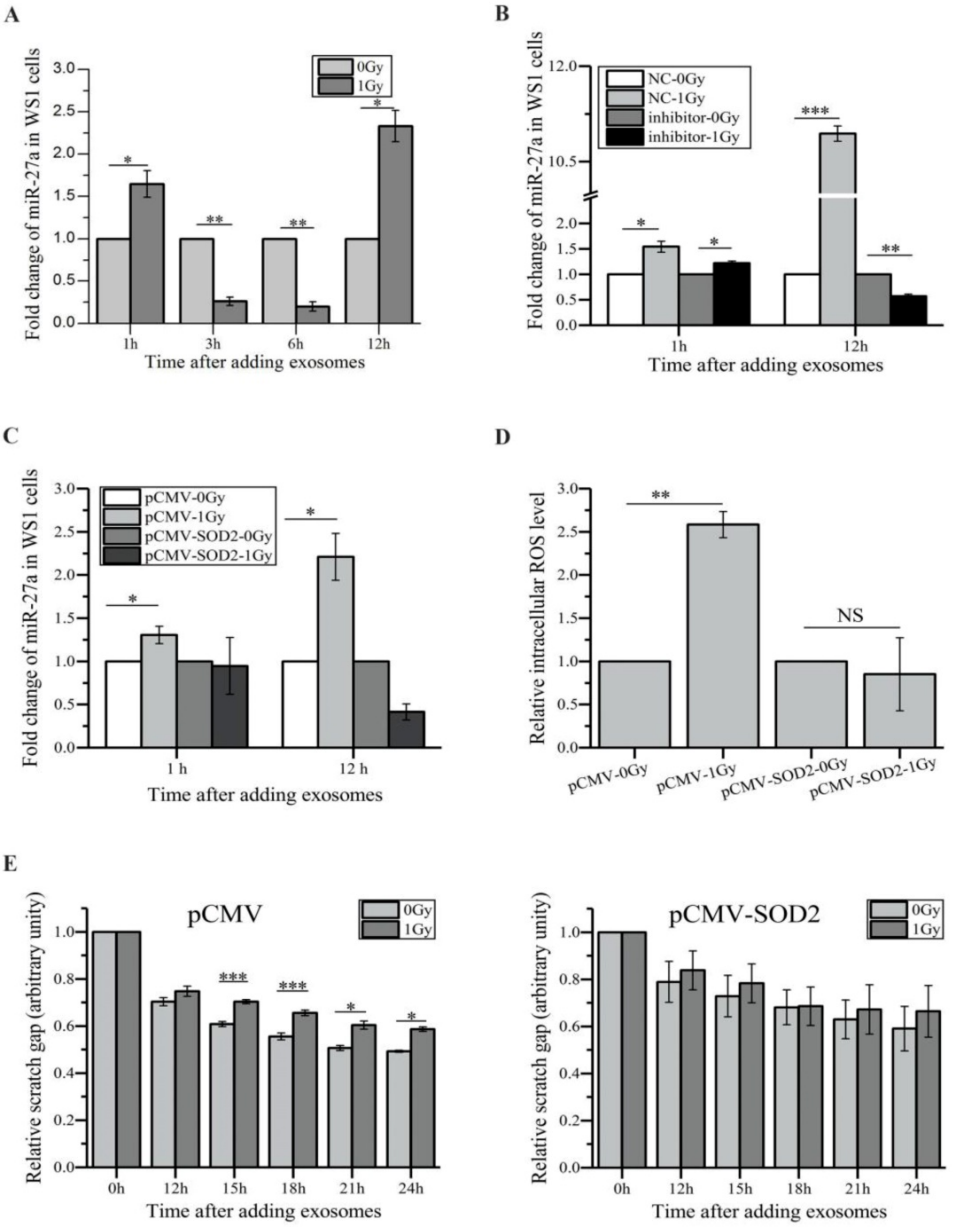
The alterations of miR-27a levels of the recipient WS1 cells after culture with exosomes and the effects of the redox status of recipient WS1 cells. (A) The dependence of the alterations of the miR-27a levels of the recipient WS1 cells on the culture time with the 3 h exosomes. (B) The alterations of the miR-27a levels of the recipient WS1 cells after culture with the 3 h exosomes from the HaCaT cells pre-transfected with miR-27a inhibitors and NC. (C) No increase in the miR-27a levels of the recipient WS1 cells overexpressing SOD2 was observed after culture with the 3 h exosomes from irradiated HaCaT cells. (D) No elevation of the intracellular ROS levels was observed in the recipient WS1 cells overexpressing SOD2 after culture with the 3 h exosomes from irradiated HaCaT cells. (E) No significant reduction in the migration rate was observed in the recipient WS1 cells overexpressing SOD2 after culture with the 3 h exosomes from irradiated HaCaT cells. All the data represent the means ± SEM from three independent experiments (n=3). *P<0.05, **P<0.01 and ***P<0.001 compared with the relative control. NS, not significant.

We next investigated how exosome-encapsulated miR-27a secreted by irradiated HaCaT cells induced bystander effects in unexposed WS1 cells. Compared with the WS1 cells cultured with the exosomes from unirradiated HaCaT cells, culturing WS1 cells with the exosomes collected from irradiated HaCaT cells 3 h post radiation for 1 and 12 h induced a 1.6-fold and 2.3-fold increase in miR-27a expression levels of WS1 cells, respectively, although there was a decrease when culturing for 3 and 6 h (Figure 5A). On the contrary, only slight increase was observed in the recipient WS1 cells after 1 h of culture with the exosomes from irradiated HaCaT cells pre-transfected with miR-27a inhibitors, and no increase was detected after 12 h of culture (Figure 5B). Moreover, there was no increase in the miR-27a levels in the SOD2-overexpressed WS1 cells after culture with the 3 h exosomes from irradiated HaCaT cells (Figure 5C). These results agreed with that no significant bystander effects were observed when irradiated HaCaT cells were deficient with miR-27a or when the recipient WS1 cells were overexpressing SOD2 (Figure 3J, K), Figure 4G, H), Figure 5D, E). We have already found that overexpression of miR-27a in WS1 cells caused bystander-like effects, e.g. elevated ROS levels and slowed migration (Figure 3E, F). Therefore, it was very likely that the miR-27a-containing exosomes from irradiated HaCaT cells induced bystander effects in unexposed WS1 cells via up-regulating their miR-27a expression levels, which was dependent on the redox status of WS1 cells.

**Figure 6:**
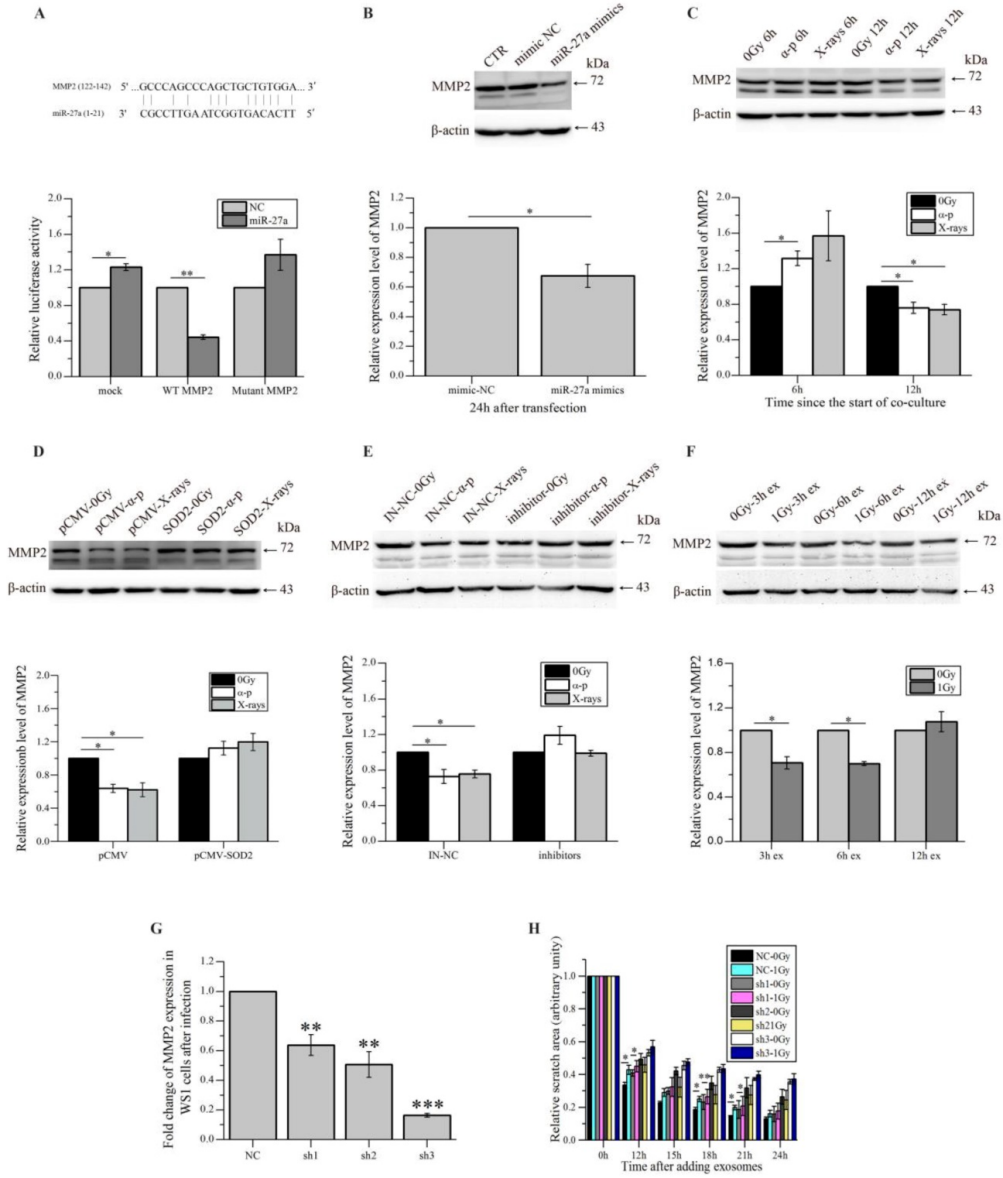
miR-27a acts through MMP2 to exert bystander effects. (A) Potential miR-27a binding site in the 5'-UTR of MMP2 and the effects of the binding site on luciferase activity. (B) The representative western blot image and the quantification of the levels of MMP2 expression in WS1 cells transfected with miR-27a mimics and NC. (C) The representative western blot image and the quantification of the levels of MMP2 expression in bystander WS1 cells after co-culture with unirradiated and irradiated HaCaT cells for 6 and 12 h. (D) The representative western blot image and the quantification of the levels of MMP2 expression in bystander WS1-pCMV and WS1-pCMV-SOD2 cells after co-culture with unirradiated and irradiated HaCaT cells for 12 h. (E) The representative western blot image and the quantification of the levels of MMP2 expression in bystander WS1 cells after co-culture with unirradiated and irradiated HaCaT cells pre-transfected with miR-27a inhibitors and NC for 12 h. (F) The representative western blot image and the quantification of the levels of MMP2 expression in recipient WS1 cells after culture with the exosomes from unirradiated and irradiated HaCaT cells collected at different times post radiation for 12 h. (G) The MMP2 mRNA expression levels in WS1 cells after infection with lentiviral particles containing three MMP2-targeting shRNAs. (H) The quantification of the area of the wound scratches of the recipient MMP2-downregulated WS1 cells after culture with the 3 h exosomes from unirradiated and irradiated HaCaT cells. All the data represent the means ± SEM from three independent experiments (n=3). *P<0.05, **P<0.01 and ***P<0.001 compared with the relative control.

To further explore how miR-27a up-regulation in WS1 cells caused slowed cell migration, we tried to identify the target gene of miR-27a, which affects cell migration. By using bioinformatics analysis (http://www.mirbase.org/index.shtml), MMP2 that has been shown to promote cell migration 46 was identified as a potential target of miR-27a, and the 5'-UTR of MMP2 contains a potential miR-27a binding site (GCCCAGCC CAGCTGCTGTGGA) (Figure 6A). Then fluorescent reporter assay was then performed to verify that MMP2 is indeed a direct target of miR-27a. As shown in the lower panel of Figure 6A, compared with the negative control (mock), transfection of miR-27a mimics significantly reduced the luciferase activity of the wild type MMP2 5'-UTR, but had no reductive effect on the luciferase activity of mutated MMP2 5'-UTR. These data indicated that miR-27a targets MMP2 through binding its 5'-UTR. Thus it was not surprising that a reduction in MMP2 expression levels was observed in the WS1 cells transfected with miR-27 mimics (Figure 6B).

Furthermore, the MMP2 expression levels of unexposed WS1 cells were found to reduce obviously after co-culture with irradiated HaCaT cells for 12 h. This reduction was not observed after shorter time (6 h) of co-culture (Figure 6C). And this reduction was also abolished in bystander WS1 cells when they were over-expressed with SOD2 (Figure 6D) and when they were co-cultured with HaCaT cells pre-transfected with miR-27a inhibitors prior to irradiation (Figure 6E). These results are all in agreement with the induction of bystander migration inhibition observed above. In addition, the exosomes from X-irradiated HaCaT cells 3 and 6 h but not 12 h post radiation induced a decrease in MMP2 expression in the recipient WS1 cells (Figure 6F), which agreed with the results above showing that the exosomes from irradiated HaCaT cells collected 3 and 6 h but not 12 h post irradiation caused migration inhibition in the unexposed recipient WS1 cells (Figure 4E).

To demonstrate that miR-27a up-regulation in WS1 cells inhibited cell migration through its direct target MMP2, we also used shRNAs to knock down the MMP2 expression of WS1 cells (Figure 6G). Not unexpectedly, the miR-27a-containing exosomes from irradiated HaCaT cells failed to induce a reduction in the cell migration rate of the recipient WS1 cells with significant lower level of MMP2 such as the WS1 cells transfected with sh2 and sh3 constructs, while the slowed cell migration was only partially inhibited in the WS1 cells transfected with sh1 constructs, which only caused MMP2 knockdown by less than 40% (Figure 6G, H).

All these data indicated that the miR-27a-containing exosomes secreted by irradiated HaCaT cells up-regulated miR-27a expression in bystander WS1 cells, elevated miR-27a in the WS1 cells then directly targeted MMP2, leading to the reduction of MMP2 expression, thus delayed cell migration.

### Exosomes secreted by irradiated HaCaT cells inhibit wound healing *in vivo*

**Figure 7:**
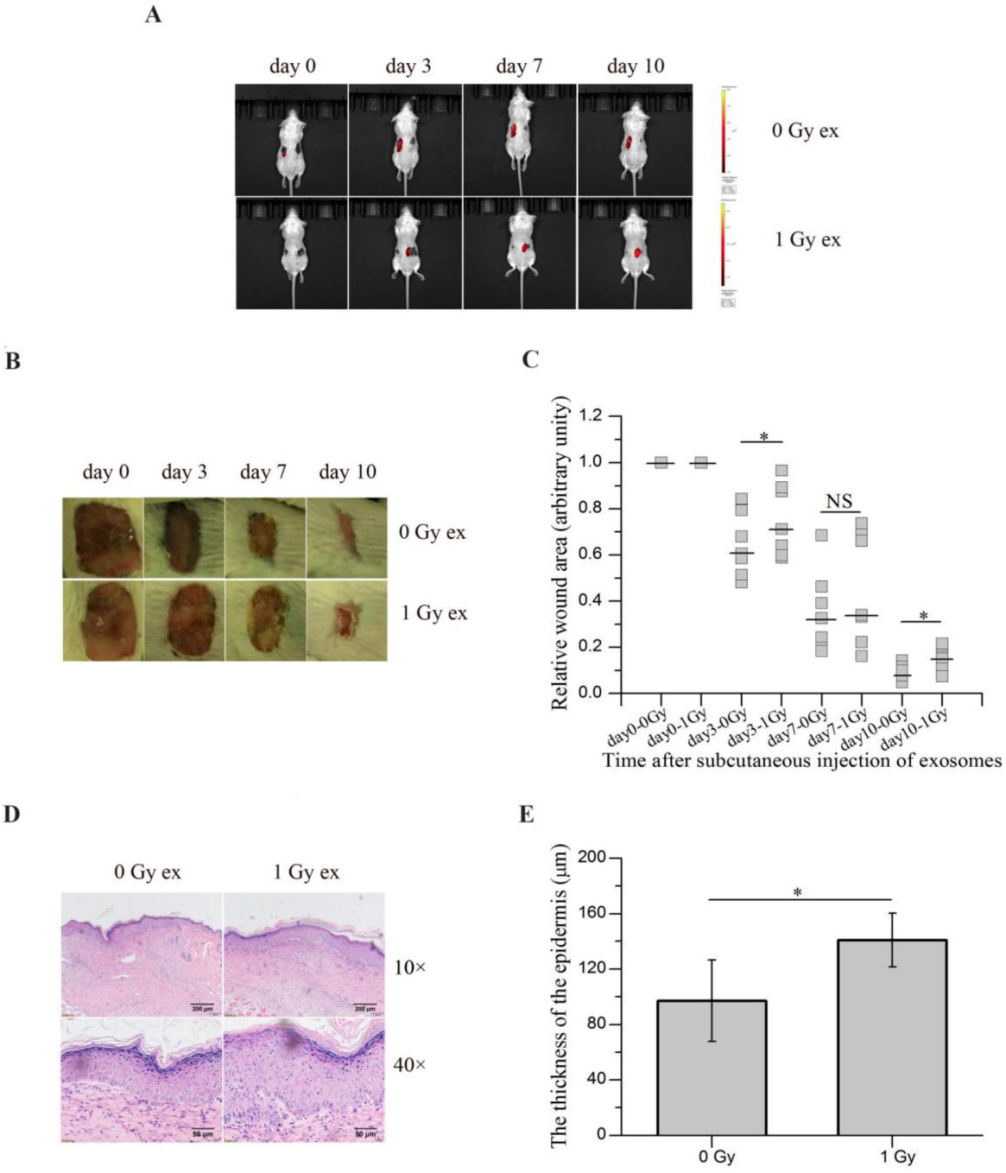
The exosomes secreted by irradiated HaCaT cells inhibit wound healing in vivo. (A) in vivo tracking of DiI-labelled exosomes (red) in mouse on different days after exosome injection. (B) The representative images of the wounds on both sides of the lower back of the same mouse on different days after subcutaneous injection of the exosomes from unirradiated and irradiated HaCaT cells. (C) The quantification of the size of the wounds on both sides of the lower back of the same mouse on different days after subcutaneous injection of exosomes from unirradiated and irradiated HaCaT cells (n=7). (D) The representative H&E staining images of the epidermis growing back in the wound areas after subcutaneous injection of the exosomes from unirradiated and irradiated HaCaT cells, showing thicker epidermis in wound area subcutaneously injected with exosomes from irradiated HaCaT cells. (E) The quantification of the thickness of epidermis growing back in the wound areas after subcutaneous injection of exosomes from unirradiated and irradiated HaCaT cells. The data represent the means ± SE from 7 mice (n=7). *P<0.05 compared with the relative control. NS, not significant.

To evaluate the potential contribution of RIBEs in wound healing, we subcutaneously injected the exosomes released from irradiated HaCaT cells near the wounds of BALB/c mice and monitored the wound healing. Since the exosomes from unirradiated and irradiated HaCaT cells were tested on the same mouse, one kind of exosomes on each side, it was important to ensure that the exosomes from unirradiated and irradiated cells did not transferred to another side and interfered with each other. Thus the exosomes were labelled with DiI prior to injection, the bioluminescence imaging showed diffused DiI fluorescence along with time. And no fluorescence signal was detected on another side where unlabelled exosomes were injected (Figure 7A), confirming that the exosomes injected on one side of mice would not affect another side. As shown in Figure 7B, C, injection of the exosomes from irradiated HaCaT cells 3 h post radiation delayed wound healing of mice compared with injection of the exosomes from unirradiated HaCaT cells. Moreover, the epidermis was found to be thickened on the 10^th^ day after injection on the side where the exosomes from irradiated keratinocytes were injected compared with the control side (Figure 7D, E). All of these results indicated that the exosomes derived from irradiated keratinocytes could inhibit wound healing *in vivo*, suggesting a potential role of RIBEs in wound healing.

## Discussion

Radiation-induced bystander effect has been widely accepted as a new dogma of radiation biology. Its occurrence expands radiation-induced damage to the outside area of radiation field, thus it may play a critical role in the health risk in individuals exposed to IR and the efficacy and the side effects of radiotherapy for cancer patients. Although the mechanisms underlying RIBEs have been intensively investigated for more than 25 years, the regulatory effects of miRNAs on RIBEs are still poorly understood. Limited studies have shown that RIBEs can be mediated by some specific miRNAs such as miR-21, miR-1246, miR-7-5p, miR-663, etc. 17, 24-26, 29, 30. Our previous studies have revealed an important role of miR-21 in the occurrence of bystander DNA damage and chromosome aberration in the HaCaT-WS1 two cell line-co-culture system 27, 28. In the present study, using the same system we first confirmed oxidative stress in bystander WS1 cells after co-culture with HaCaT cells irradiated with both α-particles and X-rays, which we have previously reported 27. We also found that the migration of bystander WS1 cells was inhibited, which was independent on cell proliferation (Figure 1), indicating a bystander inhibitory effect on cell migration. We then continued to explore whether miRNAs were involved in the bystander migration inhibition, which may affect tissue repair 40.

We identified miR-27a as a major bystander factor that induced inhibitory effect on bystander cell migration. A previous study found no significant change in miR-27a levels in irradiated cells 24 h post γ-irradiation 47. However, we observed a dramatic increase in the miR-27a levels in irradiated HaCaT cells 1 h post α- and X-irradiation compared with the sham control (Figure 3H). Moreover, the increase was critical for the induction of bystander effects, since transfection of miR-27a inhibitors into HaCaT cells prior to radiation abolished the bystander oxidative stress and migration inhibition in unirradiated WS1 cells (Figure 3J, K). Then we hypothesized that irradiated HaCaT cells with elevated miR-27 levels secreted redundant miR-27a into the co-culture media shortly after IR to induce the bystander effects. Interestingly, we also observed that the elevated miR-27a levels in irradiated HaCaT cells decreased along with time (Figure 3H), which was in support of our hypothesis.

Exosomes can act as a means of intercellular communication 31. It has been found that irradiated HaCaT cells can release exosomes to mediate communication between irradiated and bystander cells 48. Therefore, we isolated exosomes from HaCaT cells, and found an increase in the miR-27a levels in the exosomes isolated 3 and 6 h but not 12 h after IR from irradiated HaCaT cells compared with those from sham-irradiated HaCaT cells (Figure 4B). Moreover, uptake of the 3 and 6 h exosomes but not 12 h exosomes by WS1 cells induced bystander-like effects, such as the rise of intercellular ROS levels and slowed cell migration (Figure 4C, D, E). Additionally, no increase in miR-27a concentration was observed in the 3 h exosomes isolated from irradiated HaCaT cells pre-transfected with miR-27a inhibitors (Figure 4F). And these exosomes failed to induce statistically significant bystander-like effects, although there was still a trend (Figure 4G, H). All these data indicated that the miR-27a-containing exosomes released by irradiated HaCaT cells were a major mediator of the intercellular communication between irradiated HaCaT cells and unirradiated WS1 cells, leading to oxidative stress and inhibited cell migration in WS1 cells. It was similar to what Xu etc. 30 and Song etc. 25 found that exosome-mediated microRNA transfer mediated RIBEs. Meanwhile, we did not rule out the role of other RIBE factors. Culturing WS1 cells with the exosomes with low miR-27a level still induced slight oxidative stress and elevation of miR-27a expression in WS1 cells shortly after treatment (Figure 4G, Figure 5B). Moreover, the cells also showed a reduction trend in cell migration rate, although there was no statistical significance compared with the relative control (Figure 4H). These data suggested the existence of RIBE factors other than miR-27a released by irradiated HaCaT cells.

The induction of medium-mediated RIBEs requires three steps e.g. the intracellular signal transduction in irradiated cells leading to the release of bystander signaling molecules, the transport of bystander signaling molecules and the intracellular signal transduction in bystander cells upon receiving bystander signals resulting in the expression of bystander response. After identifying miR-27a as a major signaling molecule that was transported from irradiated HaCaT cells to unirradiated WS1 cells via exosomes, we also explored how it led to the observed RIBEs in the recipient WS1 cells. We found that the miR-27a levels in the recipient WS1 cells were increased upon the uptake of exosomes from irradiated HaCaT cells. But unexpectedly, this elevation did not occur persistently (Figure 5A). We are not able to provide a rational explanation for the decrease in miR-27a levels in WS1 cells after incubation with the exosomes from irradiated HaCaT cells for 3 and 6 h yet at this time, but the exosomal miR-27a did lead to a significant increase in miR-27a levels in the recipient WS1 cells after 1 and 12 hours of incubation, which were consistent with the time when ROS level elevation and slowed cell migration were observed, respectively. Furthermore, we experimentally validated MMP2, which is associated with cell migration 46, as a direct target of miR-27a (Figure 6A). In agreement with that, we observed a decrease in MMP2 expression in unirradiated WS1 cells co-cultured with irradiated HaCaT cells and incubated with the 3 h and 6 h exosomes from irradiated HaCaT cells (Figure 6C, F). Moreover, by knocking down the MMP2 gene in WS1 cells, we found that the slowed migration of WS1 cells after incubation with 3 h exosomes from irradiated HaCaT cells was significantly attenuated (Figure 6G, H). All these data indicate that co-culturing with irradiated HaCaT cells causes an elevation of the miR-27a levels in bystander WS1 cells, then leads to the inhibition of cell migration via targeting MMP2.

Moreover, the early oxidative stress in the bystander WS1 cells after co-culture with irradiated HaCaT cells may also contribute to the inhibition of bystander WS1 cell migration. We found that when abolishing the oxidative stress in the bystander WS1 cells, the WS1 cells no longer displayed the elevation of miR-27a expression and slowed migration (Figure 1E, 2B, 5C, E). In agreement with that, abolishing oxidative stress also abolished the reduction trend in MMP2 expression in the bystander WS1 cells (Figure 6D). As elevation of miR-27a level could cause oxidative stress in WS1 cells vice versa (Figure 3E), these data suggest that the cellular redox status and miR-27a expression may regulate mutually. Most importantly, the occurrence of radiation-induced bystander inhibitory effect on WS1 cell migration seems dependent on the redox status of WS1 cells. In addition to miR-27a-MMP2 pathway, ROS may also contribute to bystander migration inhibition through other pathways 49, 50.

Interestingly but not unexpectedly, we observed a prolonged wound healing and thickened epidermis in mice after subcutaneous injection of the exosomes isolated from irradiated HaCaT cells (Figure 7). This suggests that irradiated cells might release some factors that could affect tissue microenvironment leading to the interference of tissue repair and remodeling, which supports the hypothesis that RIBEs may affect wound healing process 39.

**Figure 8:**
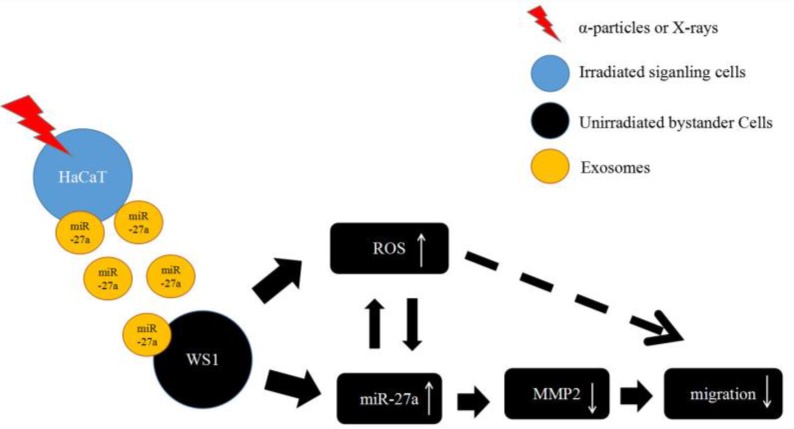
The proposed working model for the slowed cell migration of unirradiated bystander WS1 cells after co-culture with irradiated HaCaT cells.

To summarize, the present study has revealed a critical role of miR-27a in every step of the induction of the bystander migration inhibition of unirradiated WS1 fibroblasts after co-culture with irradiated HaCaT keratinocytes, confirming the important regulatory effects of miRNA on RIBEs. More specifically, the elevation of miR-27a expression in irradiated HaCaT cells is essential for the induction of the bystander effect, miR-27a-containing exosomes is a major bystander signaling factor, and the regulatory function of miR-27a-MMP2 in bystander WS1 cells is required for the inhibition of cell migration. Moreover, the occurrence of miR-27a-MMP2-regulated migration inhibition of bystander cells depends on the intracellular oxidative stress status (Figure 8). These results may have an important implication in various scenarios such as skin damage repair in individuals who are exposed to IR by accident and cancer patients who receive radiation therapy.

## Supplementary Material

Supplementary figures and tables.

## References

[1] Nagasawa H, Little JB (1992). Induction of sister chromatid exchanges by extremely low doses of alpha-particles. Cancer Res.

[2] Azzam EI, de Toledo SM, Little JB (2001). Direct evidence for the participation of gap junction-mediated intercellular communication in the transmission of damage signals from alpha-particle irradiated to nonirradiated cells. Proc Natl Acad Sci USA.

[3] Yang H, Asaad N, Held KD (2005). Medium-mediated intercellular communication is involved in bystander responses of X-ray-irradiated normal human fibroblasts. Oncogene.

[4] Prise KM, Belyakov OV, Folkard M (1998). Studies of bystander effects in human fibroblasts using a charged particle microbeam. Int J Radiat Biol.

[5] Zhou H, Randers-Pehrson G, Waldren CA (2000). Induction of a bystander mutagenic effect of alpha particles in mammalian cells. Proc Natl Acad Sci USA.

[6] Harada K, Nonaka T, Hamada N (2009). Heavy-ion-induced bystander killing of human lung cancer cells: role of gap junctional intercellular communication. Cancer Sci.

[7] Kadhim M, Salomaa S, Wright E (2013). Non-targeted effects of ionising radiation-implications for low dose risk. Mutat Res.

[8] Prise KM, O'Sullivan JM (2009). Radiation-induced bystander signalling in cancer therapy. Nat Rev Cancer.

[9] Lara PC, López-Peñalver JJ, Farias Vde A (2015). Direct and bystander radiation effects: a biophysical model and clinical perspectives. Cancer Lett.

[10] Edwards GO, Botchway SW, Hirst G (2004). Gap junction communication dynamics and bystander effects from ultrasoft X-rays. Br J Cancer.

[11] Sasi SP, Song J, Park D (2014). TNF-TNFR2/p75 signaling inhibits early and increases delayed nontargeted effects in bone marrow-derived endothelial progenitor cells. J Biol Chem.

[12] Desai S, Kumar A, Laskar S (2013). Cytokine profile of conditioned medium from human tumor cell lines after acute and fractionated doses of gamma radiation and its effect on survival of bystander tumor cells. Cytokine.

[13] Shao C, Folkard M, Prise KM (2008). Role of TGF-beta1 and nitric oxide in the bystander response of irradiated glioma cells. Oncogene.

[14] Iyer R, Lehnert BE, Svensson R (2000). Factors underlying the cell growth-related bystander responses to alpha particles. Cancer Res.

[15] Peng Y, Zhang M, Zheng L (2017). Cysteine protease cathepsin B mediates radiation-induced bystander effects. Nature.

[16] Xiao L, Liu W, Li J (2014). Irradiated U937 cells trigger inflammatory bystander responses in human umbilical vein endothelial cells through the p38 pathway. Radiat Res.

[17] Jiang Y, Chen X, Tian W (2014). The role of TGF-β1-miR-21-ROS pathway in bystander responses induced by irradiated non-small-cell lung cancer cells. Br J Cancer.

[18] Azzam EI, De Toledo SM, Spitz DR (2002). Oxidative metabolism modulates signal transduction and micronucleus formation in bystander cells from alpha-particle-irradiated normal human fibroblast cultures. Cancer Res.

[19] Burdak-Rothkamm S, Short SC, Folkard M (2007). ATR-dependent radiation-induced gamma H2AX foci in bystander primary human astrocytes and glioma cells. Oncogene.

[20] Mothersill C, Seymour C (2012). Are epigenetic mechanisms involved in radiation-induced bystander effects?. Front Genet.

[21] Ilnytskyy Y, Koturbash I, Kovalchuk O (2009). Radiation-induced bystander effects in vivo are epigenetically regulated in a tissue-specific manner. Environ Mol Mutagen.

[22] Koturbash I, Boyko A (2007). Role of epigenetic effectors in maintenance of the long-term persistent bystander effect in spleen in vivo. Carcinogenesis.

[23] Dickey JS, Zemp FJ, Altamirano A (2011). H2AX phosphorylation in response to DNA double-strand break formation during bystander signalling: effect of microRNA knockdown. Radiat Prot Dosimetry.

[24] Yuan D, Xu J, Wang J (2016). Extracellular miR-1246 promotes lung cancer cell proliferation and enhances radioresistance by directly targeting DR5. Oncotarget.

[25] Song M, Wang Y, Shang Z (2016). Bystander autophagy mediated by radiation-induced exosomal miR-7-5p in non-targeted human bronchial epithelial cells. Sci Rep.

[26] Hu W, Xu S, Yao B (2014). MiR-663 inhibits radiation-induced bystander effects by targeting TGFB1 in a feedback mode. RNA Biol.

[27] Tian W, Yin X, Wang L (2015). The key role of miR-21-regulated SOD2 in the medium-mediated bystander responses in human fibroblasts induced by irradiated keratinocytes. Mutat Res.

[28] Yin X, Tian W, Wang L (2015). Radiation quality-dependence of bystander effect in unirradiated fibroblasts is associated with TGF-β1-Smad2 pathway and miR-21 in irradiated keratinocytes. Sci Rep.

[29] Xu S, Ding N, Pei H (2014). MiR-21 is involved in radiation-induced bystander effects. RNA Biol.

[30] Xu S, Wang J, Ding N (2015). Exosome-mediated microRNA transfer plays a role in radiation-induced bystander effect. RNA Biol.

[31] Edgar JR (2016). Q&A: What are exosomes, exactly?. Edgar BMC Biol.

[32] Jelonek K, Widlak P, Pietrowska M (2016). The influence of ionizing radiation on exosome composition, secretion and intercellular communication. Protein Pept Lett.

[33] Lehmann BD, Paine MS, Brooks AM (2008). Senescence-associated exosome release from human prostate cancer cells. Cancer Res.

[34] Hurwitz MD, Kaur P, Nagaraja GM (2010). Radiation therapy induces circulating serum Hsp72 in patients with prostate cancer. Radiother Oncol.

[35] Arscott WT, Tandle AT, Zhao S (2013). Ionizing radiation and glioblastoma exosomes: implications in tumor biology and cell migration. Transl Oncol.

[36] Mutschelknaus L, Azimzadeh O, Heider T (2017). Radiation alters the cargo of exosomes released from squamous head and neck cancer cells to promote migration of recipient cells. Sci Rep.

[37] Dinh TK, Fendler W, Chałubińska-Fendler J (2016). Circulating miR-29a and miR-150 correlate with delivered dose during thoracic radiation therapy for non-small cell lung cancer. Radiat Oncol.

[38] Ji W, Tian W, Yin X (2015). The building and validation of a novel alpha-irradiation equipment used for cultured cell study. J Radiat Res Radiat Proc.

[39] Suzuki K, Yamashita S (2014). Radiation-induced bystander response: mechanism and clinical implications. Adv Wound Care (New Rochelle).

[40] McDougall S, Dallon J, Sherratt J (2006). Fibroblast migration and collagen deposition during dermal wound healing: mathematical modelling and clinical implications. Philos Trans A Math Phys Eng Sci.

[41] Han J, Kim HJ, Schafer ST (2016). Functional Implications of miR-19 in the Migration of Newborn Neurons in the Adult Brain. Neuron.

[42] Shi DL, Shi GR, Xie J (2016). MicroRNA-27a inhibits cell migration and invasion of fibroblast-Like synoviocytes by targeting follistatin-like protein 1 in rheumatoid arthritis. Mol Cells.

[43] Lü MH, Hu CJ, Chen L (2013). miR-27b represses migration of mouse MSCs to burned margins and prolongs wound repair through silencing SDF-1a. PLoS One.

[44] Xu L, Li Q, Xu D (2014). hsa-miR-141 downregulates TM4SF1 to inhibit pancreatic cancer cell invasion and migration. Int J Oncol.

[45] Turchinovich A, Samatov TR, Tonevitsky AG (2013). Circulating miRNAs: cell-cell communication function?. Front Genet.

[46] Giannelli G, Falk-Marzillier J, Schiraldi O (1997). Induction of cell migration by matrix metalloprotease-2 cleavage of laminin-5. Science.

[47] Di Francesco A, De Pittà C, Moret F (2013). The DNA-damage response to γ-radiation is affected by miR-27a in A549 cells. Int J Mol Sci.

[48] Jella KK, Rani S, O'Driscoll L (2014). Exosomes are involved in mediating radiation induced bystander signaling in human keratinocyte cells. Radiat Res.

[49] Schröder K (2014). NADPH oxidases in redox regulation of cell adhesion and migration. Antioxid Redox Signal.

[50] Jiang C, Jiang L, Li Q (2018). Acrolein induces NLRP3 inflammasome-mediated pyroptosis and suppresses migration via ROS-dependent autophagy in vascular endothelial cells. Toxicology.

