# Supplementary material for: miR-27a-containing Exosomes Secreted by Irradiated Skin Keratinocytes Delayed the Migration of Unirradiated Skin Fibroblasts
Source: Int J Biol Sci. 2019 Aug 19;15(10):2240–55. doi: 10.7150/ijbs.35356 (PMC6775295; doi:10.7150/ijbs.35356)
Supplement: Supplementary file 1 — Supplementary figures and tables. [file ijbsv15p2240s1.pdf]

Supplementary Material

A

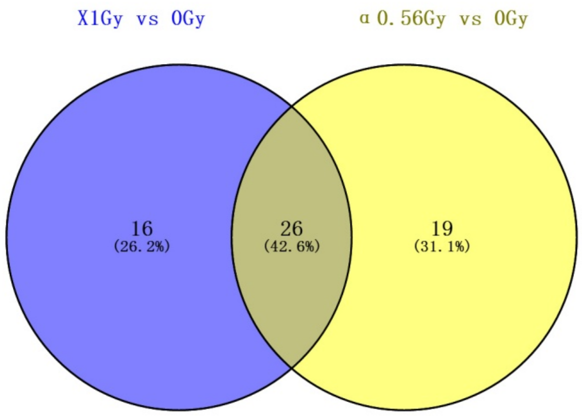

hsa-miR-141-3p
hsa-miR-151a-5p
hsa-miR-15a-5p
hsa-miR-181a-5p
hsa-miR-183-5p
hsa-miR-18a-5p
hsa-miR-19a-3p
hsa-miR-19b-3p
hsa-miR-21-3p
hsa-miR-21-5p
hsa-miR-212-3p
hsa-miR-27a-3p
hsa-miR-27b-3p
hsa-miR-29b-3p
hsa-miR-30a-3p
hsa-miR-30b-5p
hsa-miR-30c-5p
hsa-miR-31-3p
hsa-miR-34a-5p
hsa-miR-361-5p
hsa-miR-455-3p
hsa-miR-4665-3p
hsa-miR-7-5p
hsa-miR-7150
hsa-miR-8063
hsa-miR-8072

B

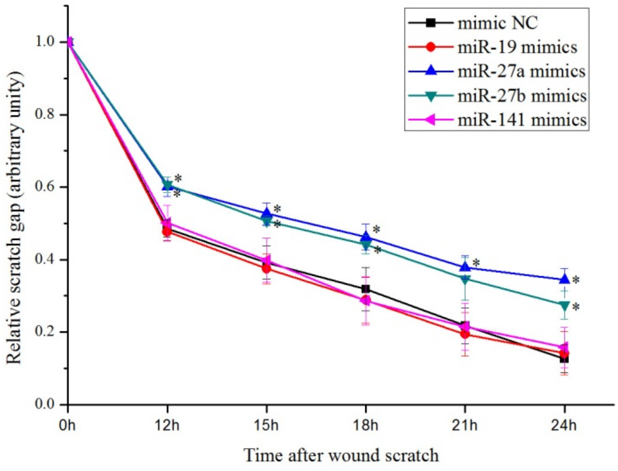

Figure S1. miR27a/b might be responsible for the slowed migration of bystander WS1 cells. (A) Identification of miRNAs in HaCaT cells up-regulated (3-fold and above

compared with unirradiated cells) by both X-irradiation and α-irradiation, which have been also reported to be associated with cell migration. Twenty-six miRNAs were found to be up-regulated in both X-irradiated and α-irradiated HaCaT cells. The list of the miRNAs is shown in the table. Among them, miR-141, miR-19a, miR-27a and miR-27b, which are highlighted in yellow, have been reported to be related to cell migration. One hour post radiation, unirradiated, 1 Gy-X-irradiated and 0.56 Gy-α-irradiated HaCaT cells were collected, total RNAs were extracted from the cells using mirVaNa^{TM} RNA Isolation Kit. miRNA Microarray was performed by Oebiotech (Shanghai, China) (n=3). (B) The effects of transfection of mimics of different miRNAs on the migration of WS1 cells, showing that overexpression of miR-27a/b but not miR-141 and miR-19a significantly delayed WS1 cell migration. WS1 cells were transfected with mimics of different miRNAs using lipofectamine® 2000 (Life Technologies, USA). (The miR-141-3p mimics, 5'-UAACACUGUC UGGUAAAGAUGGAUCUUUACCAGACAGUGUUAUU-3', miR-19a-3p, 5'-UGUGCAAAUCUAUGCAAAACUGAAGUUUUGCAUAGAUUUGCACAUU-3') Thirty-two hours after transfection, the wound scratch assay was performed on the transfected cells. All the data represent the means ± SEM from three independent experiments (n=3). *P<0.05 compared with the negative control.

0 Gy ex

			Size (d.nm):	% Number:	St Dev (d.nm):
Z-Average (d.nm):	499.2	Peak 1:	878.9	2.7	283.2
PdI:	1.000	Peak 2:	130.9	97.3	26.90
Intercept:	0.992	Peak 3:	5293	0.0	695.9

Result quality : Refer to quality report

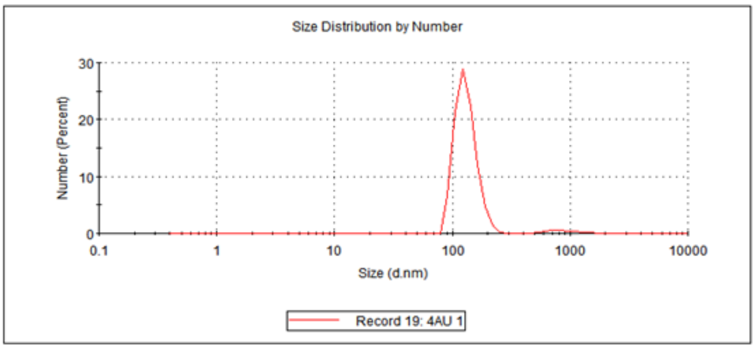

1 Gy ex

			Size (d.nm):	% Number:	St Dev (d.nm):
Z-Average (d.nm):	866.7	Peak 1:	824.5	0.4	185.9
PdI:	0.928	Peak 2:	90.35	99.6	16.69
Intercept:	1.03	Peak 3:	0.000	0.0	0.000

Result quality : Refer to quality report

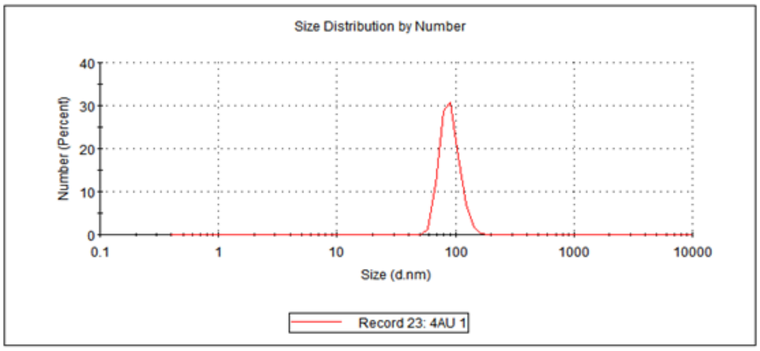

Figure S2. The size distribution of the exosomes extracted from unirradiated and X-irradiated HaCaT cells. Three hours post 1 Gy of X-irradiation, the exosomes were isolated from the media culturing unirradiated control and irradiated cells. And the size distribution was measured by Zetasizer Nano ZS 90 (Malvern, UK) (n=3).
